# Hypoxia- and Inflammation-Related Transcription Factor SP3 May Be Involved in Platelet Activation and Inflammation in Intracranial Hemorrhage

**DOI:** 10.3389/fneur.2022.886329

**Published:** 2022-06-02

**Authors:** Ding Wan, Jin Feng, Peng Wang, Zhenxing Yang, Tao Sun

**Affiliations:** ^1^Department of Neurosurgery, General Hospital of Ningxia Medical University, Yinchuan, China; ^2^Ningxia Key Laboratory of Craniocerebral Diseases, Ningxia Medical University, Yinchuan, China

**Keywords:** ICH, hypoxia, inflammation, SP3, biomarkers, cerebrovascular disease

## Abstract

The purpose of this study was to identify the biomarkers implicated in the development of intracranial hemorrhage (ICH) and potential regulatory pathways. In the transcriptomic data for patients with ICH, we identified DEmiRNAs and DEmRNAs related to hypoxia, inflammation, and their transcription factors (TFs). An ICH-based miRNA-TF-mRNA regulatory network was thus constructed, and four biomarkers (*TIMP1, PLAUR, DDIT3*, and *CD40*) were screened for their association with inflammation or hypoxia by machine learning. Following this, SP3 was found to be a transcription factor involved in hypoxia and inflammation, which regulates *TIMP1* and *PLAUR*. From the constructed miRNA-TF-mRNA regulatory network, we identified three axes, hsa-miR-940/RUNX1/TIMP1, hsa-miR-571/SP3/TIMP1, and hsa-miR-571/SP3/PLAUR, which may be involved in the development of ICH. Upregulated *TIMP1* and *PLAUR* were validated in an independent clinical cohort 3 days after ICH onset. According to Gene Set Enrichment Analysis (GSEA), SP3 was discovered to be important in interleukin signaling and platelet activation for hemostasis. Transcription factor SP3 associated with hypoxia or inflammation plays an important role in development of ICH. This study provides potential targets for monitoring the severity of inflammation and hypoxia in patients with ICH.

## Introduction

Intracranial hemorrhage (ICH) is a condition characterized by bleeding from the brain parenchyma caused by the rupture of blood vessels in the brain, which leads to compression of the surrounding nerve tissue, disruption of the brain function, and triggering of disorders (1). ICH can be triggered by various factors, such as trauma, hypertension, and infection (2, 3). ICH accounts for 10–15% of strokes and is its most lethal subtype (4–8). The formation of a hematoma from ICH can severely disrupt tracts, leading to various dysfunctions and threatening patients' lives, which makes ICH highly disabling and mortal (4). More than 1 million people are affected by ICH each year (9). The mortality rate for patients with ICH range from 30 to 50% at 1 month and 54% at 1 year (10, 11). The unprecedented virus (COVID-19) have also been identified as potential risk factors for ICH (12). Patients with ICH need to be diagnosed and treated early and accurately in order to achieve the best possible outcome.

Neuronal apoptosis, inflammation, oxidative stress, edema formation, and the breakdown of the blood–brain barrier all contribute to ICH development (13, 14). ICH is not only pathologically characterized by inflammation, but it also causes secondary damage to the brain (15, 16). Inflammatory injury can damage the vascular endothelium and, thus, is involved in ICH development (17, 18). The infiltrating leukocytes can release pro-inflammatory factors, which further damage the blood–brain barrier, thereby worsening the secondary brain injury after ICH development (19–21). Meanwhile, hypoxia can be activated through oxidative stress mechanisms, which in turn are involved in the developmental mechanisms of ICH (14, 22). Therefore, we hypothesize that inflammation and hypoxia play important roles in ICH pathogenesis; however, the molecular mechanisms involved are not yet clear.

Because inflammation is involved in secondary damage after ICH, the degree of inflammation can be used to predict the prognosis of patients with ICH (21, 23, 24). Some indicators of inflammation, such as the neutrophil-to-lymphocyte ratio, have been shown to be useful in predicting the prognosis of patients with ICH, and they are predictive of a good outcome (25). Brain tissue can become hypoxic from ICH, causing irreversible damage (26). Genes associated with hypoxia or inflammation, as well as their pathways of action, play an important role in the development and progression of ICH. ICH development is involved in the Nrf2/HO-1 signaling pathway, according to the previous studies (27). KLF6 acts as a transcription factor that mediates SIRT5 inhibition of the Nrf2/HO-1 signaling pathway, which in turn exacerbates neuronal apoptosis and oxidative stress after ICH development (28). KLF6 plays a crucial role in the inflammatory and hypoxic response (27). Inflammation and hypoxia dramatically impact the survival and quality of life of patients with ICH. Thus, identifying the degree of inflammation and hypoxia in ICH is essential for monitoring the prognosis of patients with ICH.

Recent developments in bioinformatics, including the availability of considerable RNA sequencing data resources, have provided a direction for disease diagnosis and treatment (29–32). Gene expression profiles associated with inflammation or hypoxia can be obtained from RNA sequencing data. This study was designed to examine the genes and pathways that are potentially involved in inflammation during ICH.

## Methods

### Data Downloaded From the GEO Database

The GEO database was searched for intracerebral hemorrhage-related RNA transcriptomic datasets based on the following keywords: “intracerebral haemorrhage” and “brain haemorrhage”. Exclusion criteria were set as follows: (1) transcriptomic data from animal models or knockout animals; (2) brain hemorrhage caused by vascular malformations, aneurysms, etc.; and (3) drug experiments designed. A dataset containing mixed plasma samples from 15 patients with ICH and eight healthy controls (GSE43618 dataset) was filtered based on the filtering criteria (33). Peripheral blood mRNA transcriptomic data were obtained from GSE125512 for 11 patients with ICH at the onset and 3 days after onset (34). These two datasets were used to identify differentially expressed miRNAs (DEmiRNAs) and differentially expressed mRNAs (DEmRNAs). These DEmRNAs were derived from an analysis of differences between peripheral blood transcriptome expression profiles 3 days after and at ICH onset. The transcriptome data were log-2 transformed with different unit formats, and de-batching between samples was performed. Finally, we collected peripheral blood samples from 20 patients with an ICH and 17 healthy control volunteers in order to validate the genes related to inflammation and hypoxia identified by the bioinformatics analysis. Included patients must meet the following criteria: (1) experience acute cerebral hemorrhage within 3 days; (2) have clear diagnostic imaging and laboratory results; (3) have no vascular malformations, coagulation disorders, or other causes of bleeding; and (4) be free of malignant tumors and other serious diseases. Ethics approval for this study was obtained from the General Hospital of Ningxia Medical University. All the participants provided written informed consent.

### Inflammation or Hypoxia-Associated Gene Sets

In order to select a broad list of candidate gene sets for inflammation- and hypoxia-related genes, we searched the Kyoto Encyclopedia of Genes and Genomes (KEGGs) database (www.kegg.jp). Furthermore, the PubMed and Web of Science databases were searched for inflammation- or hypoxia-related gene complements. Ultimately, a total of 50 hypoxia-related genes and 200 inflammation-related genes were identified. These genes are mainly involved in inflammatory or hypoxic response processes during disease development.

### Analysis of Variances

First, the limma R package was used to identify DEGs, including DEmiRNAs and DEmRNAs, in the occurrence of ICH (35). We identified mRNAs that are up- and down-regulated in patients with ICH 3 days after cerebral hemorrhage compared with those at the onset of cerebral hemorrhage using differential expression analysis of the peripheral blood transcriptome. DEGs were filtered using *p* < 0.05 as the threshold. Data were then filtered by genes using Perl (https://www.perl.org/) to obtain dysregulated genes associated with inflammation or with hypoxia and the corresponding gene expression matrix.

### Biological Functional Pathway Analysis

Functional pathway analysis of up- or down-regulated DEmRNAs using the Kyoto Encyclopedia of Genes and Genomes (KEGGs) and Gene Ontology (GO). GO terminology is described in three parts: biological processes (BPs), cellular components (CCs), and molecular functions (MFs). The clusterProfiler R package was used to complete GO and KEGG analysis as previous research (36, 37). Further, GSEA is used to analyze functional pathways of dysregulated biology involving key genes (38). Selected functional pathways for differential analysis are referenced from “c2.cp.v7.2.symbols.gmt [Curated]” in MSigDB collections (https://www.gsea-msigdb.org/gsea/msigdb/) gene set. The threshold used to identify dysfunctional pathways was set at a false discovery rate of <0.25 and adjusted *p* of <0.05.

### Construction of miRNA–TF–mRNA Network

According to the previous study, the miRNA–TF–mRNA network was constructed (39–42). First, TF–mRNA relationship pairs were predicted in DEmRNAs using the TRRUST (v2) database (43), where mRNAs were associated with hypoxia or inflammation. To explore regulatory relationships between DEmiRNAs and DEmRNAs (including TFs), miRWalk was used (http://mirwalk.umm.uni-heidelberg.de/>). R software was used to match the interrelationships between DEmiRNAs and DEmRNAs. Among the predicted miRNA–mRNA molecular pairs, only those with opposite regulatory directions were subjected to further analysis. Furthermore, Cytoscape was used to visualize the entire miRNA–TF–mRNA interaction network and to identify the hub genes in the network based on the number of connections in each node (44).

### Machine Learning

Intelligent machines are converging with advancing biotechnologies to shape the future of medicine (45). Machine learning is used to screen genes associated with the progression of ICH. Support Vector Machine–Recursive Feature Elimination (SVM–RFE) was used to investigate genes associated with hypoxia and inflammation (46). SVM is excellent at handling small datasets and shows good classification performance. Redundant genes are filtered using the iterative algorithm of SVM–RFE, resulting in genes highly correlated with the outcome. Furthermore, the least absolute shrinkage and selection operator (LASSO) is used for gene screening. As previous research, LASSO analysis was performed using the “glmnet” R package (42, 47). ROC curves were used to assess the predictive ability of core genes to distinguish between patients with ICH at different progression stages, thereby testing their reliability for the outcome prediction. Principle component analysis and *t*-distributed stochastic neighbor embedding (t-SNE) (48) were used to demonstrate the ability of screened core genes to classify patients with ICH at different developmental stages. The R package “Rtsne” was used to implement the *t*-SNE algorithm based on non-linear dimensionality reduction.

### Quantitative qRT-PCR

Total RNA was extracted using TRIzol (TaKaRa Bio, Shiga, Japan) and reverse-transcribed into cDNA using PrimeScript RT Master Mix (TaKaRa Bio, Shiga, Japan). According to the manufacturer's instructions, Real-time PCR was performed using SYBR Green PCR Master Mix (Takara). Using GAPDH as a reference, the 2^−Δ*ΔCt*^ method was applied for the relative quantification of core gene expression levels and normalized.

### Statistical Analysis

All the drawings are performed using the R software (version 4.0.5). The “VennDiagram” R package is used to create a Venn diagram to present the results of the gene intersection analysis. Spearman's correlation test was used to assess the correlation between key genes, and correlation coefficients of >0.3 were considered to be co-expression relationships. Differential analysis of gene expression between the two groups was performed using the Wilcoxon rank-sum test. Unless otherwise indicated, *p* < 0.05 was considered a statistically significant difference.

## Results

### Neutrophils Play an Important Role in ICH Development

**Figure 1:**
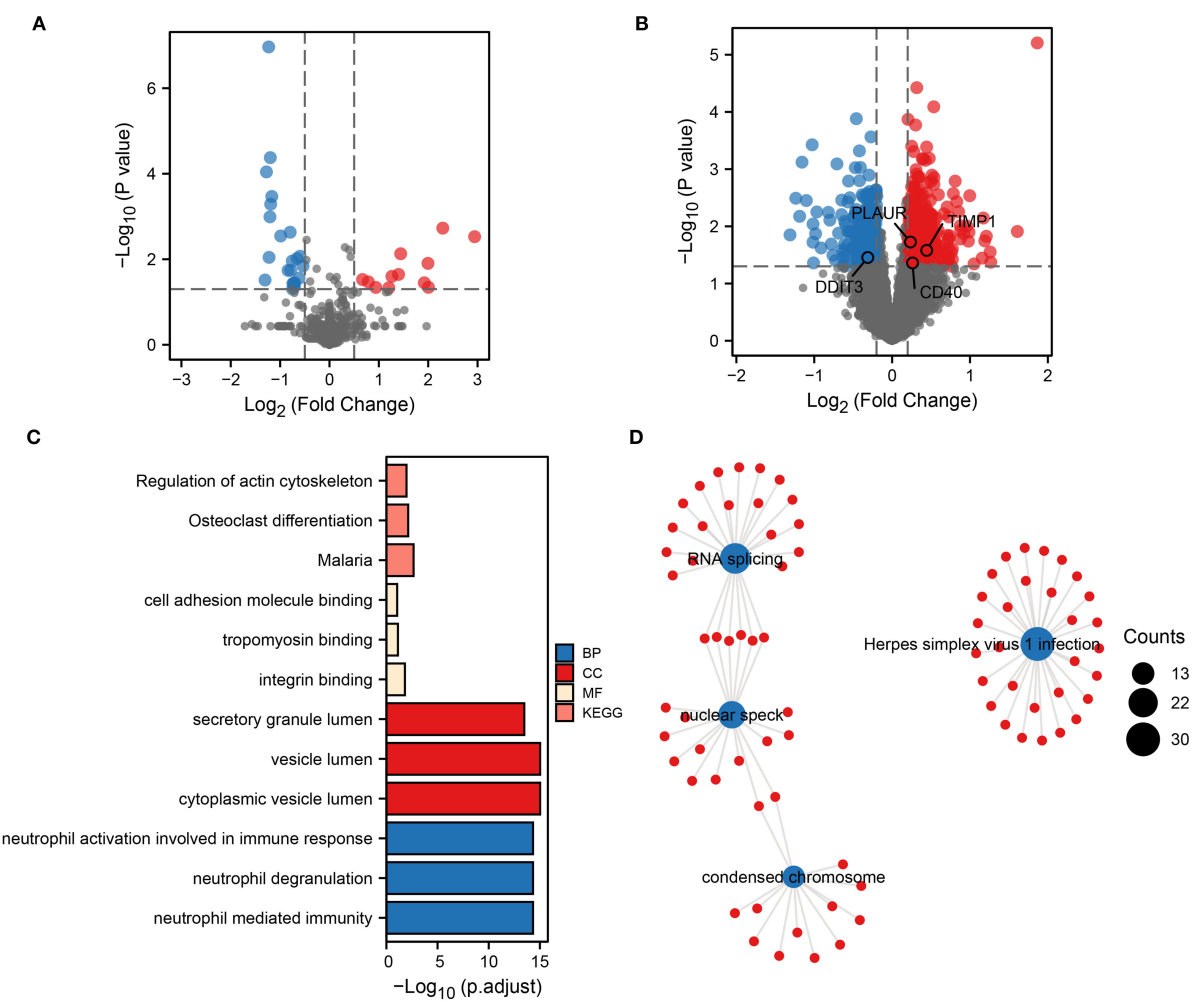
Biological functional pathways of DEGs. Volcano plot showing DEmiRNAs **(A)** and DEmRNAs **(B)**. Red represents upregulated genes, and blue represents downregulated genes. Upregulated **(C)** and downregulated **(D)** biological functional pathways enriched by DEGs.

To obtain biomarkers and pathways associated with the occurrence or progression of ICH, we performed a differential expression analysis. First, 46 DEmiRNAs were identified by differential analysis between ICH and healthy control (HC) (Figure 1A). Based on the transcriptomic data obtained from the peripheral blood of ICH at different development stages, 914 DEmRNAs were identified. Compared to the onset of ICH, 444 genes were upregulated and 470 genes were downregulated 3 days after the onset of ICH (Figure 1B). These up- and downregulated genes were then subjected to separate functional pathway analyses. The main pathways shown to be upregulated in ICH by GO and KEGG included neutrophil-mediated immunity, secretory granule lumen, cell adhesion molecule binding, and regulation of actin cytoskeleton (Figure 1C). In addition, the pathways enriched by the downregulated genes contained RNA splicing, nuclear speck, Herpes simplex virus 1 infection, and condensed chromosome (Figure 1D). These results suggest that neutrophils play an important role in the development of ICH.

### Identification of Differentially Expressed Genes Associated With Inflammation or Hypoxia

**Figure 2:**
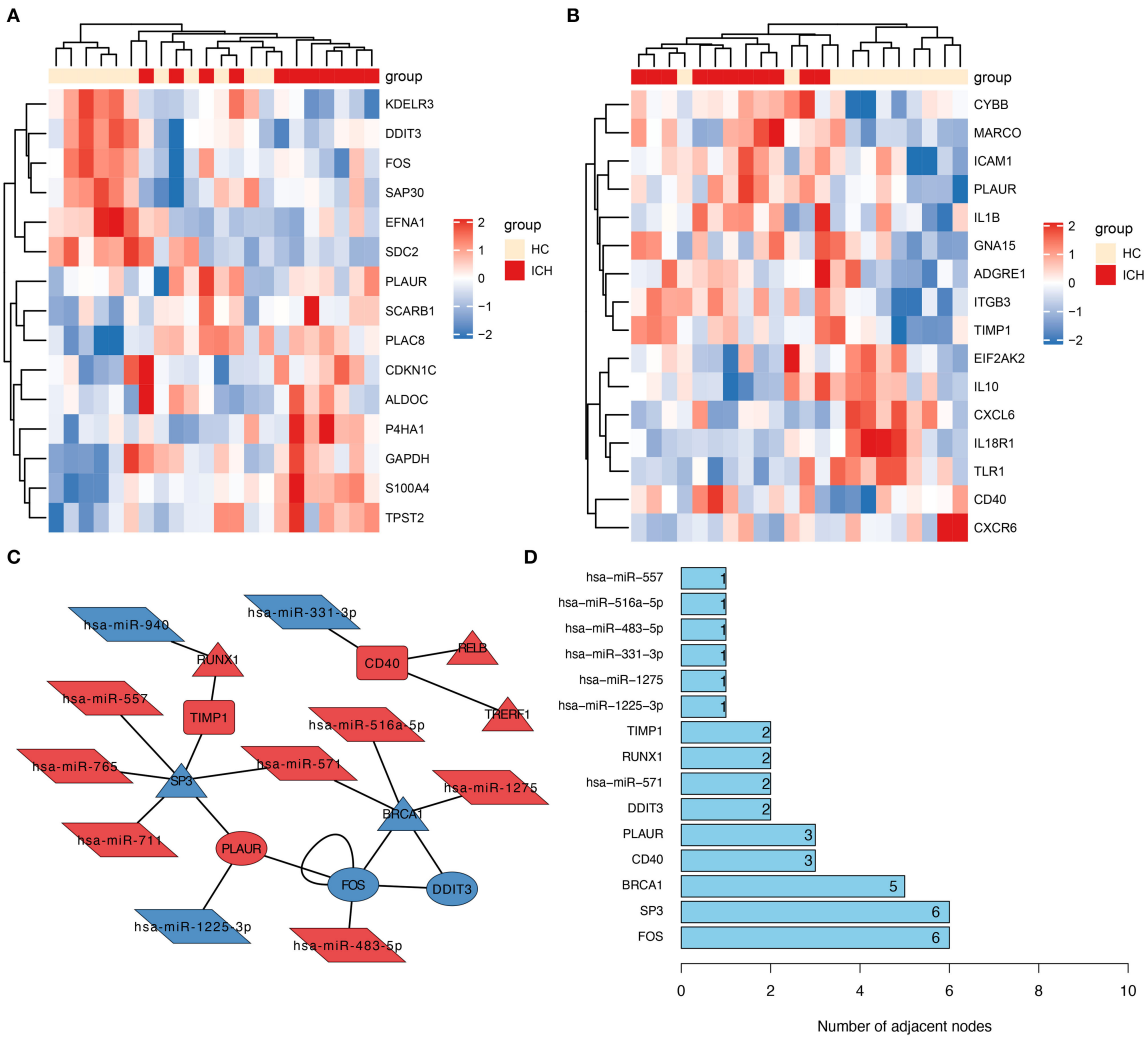
Construction of molecular interactions networks. Heat map showing hypoxia- **(A)** and inflammation-related **(B)** DEGs in patients with ICH. The depth of the color was used to indicate the intensity of gene expression. **(C)** The constructed miRNA–TF–mRNA network. Each node represents a gene and each edge represents a gene interaction. Triangle represents transcription factors, parallelogram represents miRNAs, ellipse represents hypoxia-related genes, and the round rectangle represents inflammation-related genes. **(D)** Number of junctions between individual genes in this miRNA–TF–mRNA network.

To explore the possible role of inflammation- or hypoxia-related genes in ICH development, we further screened the inflammation- and hypoxia-related genes separately in the DEmRNAs. We identified 15 genes associated with hypoxia; their relative expression between ICHs is shown in Figure 2A. The number of genes related to inflammation was 16 (Figure 2B). The corresponding heat map showed a clear boundary between hypoxia or inflammation-related DEGs in patients with different stages of ICH. This suggests that the genes associated with hypoxia or inflammation play an important role in ICH development.

### PPI Network Shows SP3 as a Hub Gene and May Be Associated With Hypoxia and Inflammation

From all the DEmRNAs, eight differentially expressed transcription factors (DETFs) were identified, which can regulate some of these genes in DEGs associated with hypoxia or inflammation. Furthermore, 10 DEmiRNAs were predicted to potentially act on these DEmRNAs (or DETFs) associated with hypoxia or inflammation. And the relationship pairs between these DEmiRNAs and DEmRNAs (including DETFs) were constructed as an miRNA–TF–mRNA interaction network (Figure 2C). From this molecular interaction network, SP3 was identified as the hub gene in the network, as it has the highest number of linkages (Figure 2D). From the constructed molecular interaction network, we further identified a possible correlation between *SP3* and *TIMP1* and *PLAUR*. *TIMP1* is an inflammation-related gene, while *PLAUR* is a gene associated with hypoxia and inflammation. Therefore, the hub gene *SP3* might be a transcription factor associated with hypoxia and inflammation.

### Thirteen Important Genes Obtained From Machine Learning Screening

**Figure 3:**
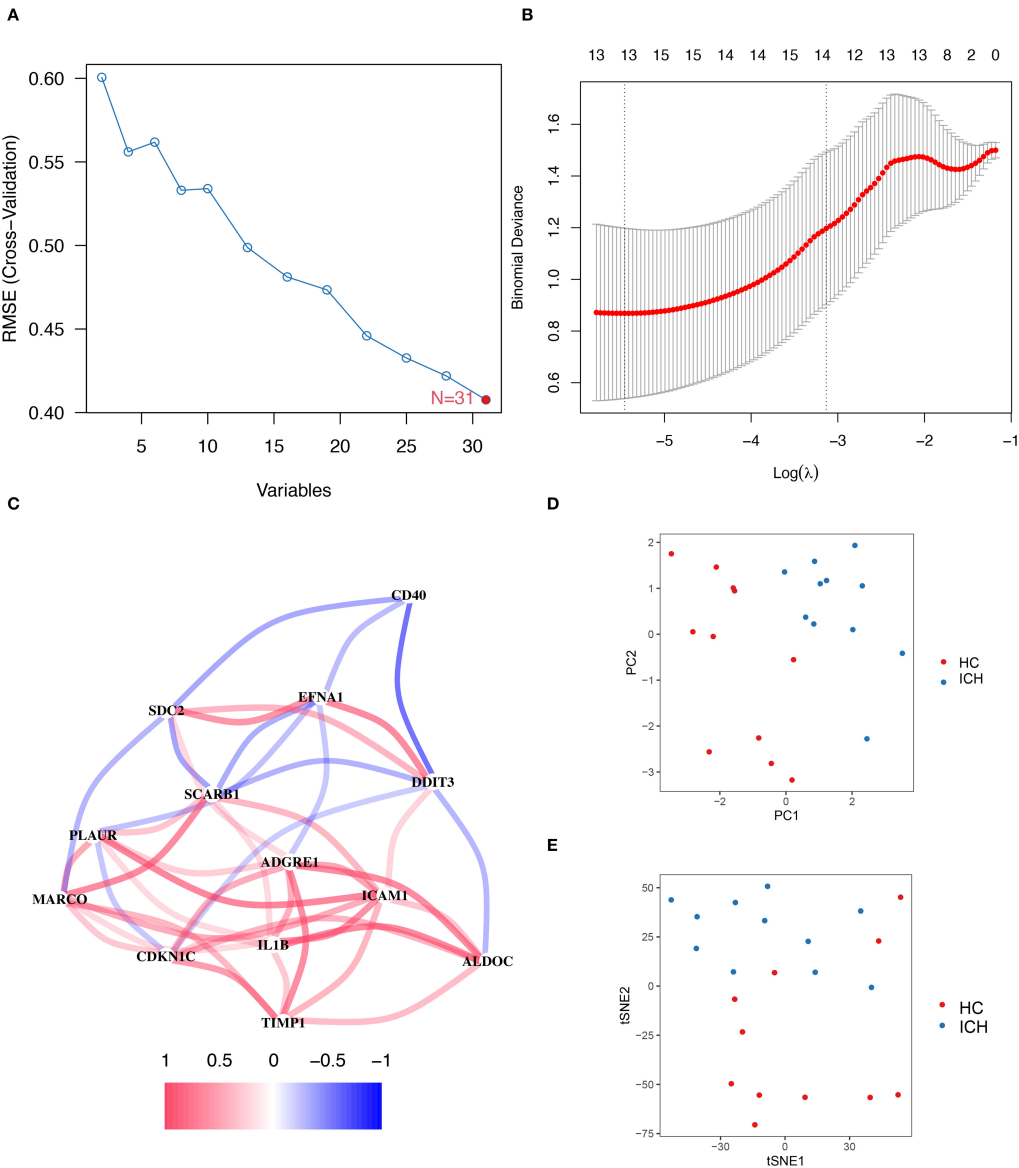
SVM–RFE and LASSO regression analysis results. **(A)** On a line graph the prediction accuracy of each variable included in the model is displayed. And the SVM–RFE screening process showed the smallest errors were obtained when all 31 genes were included in the model. **(B)** The LASSO analysis resulted in a final screening of 13 genes. **(C)** Correlation linkage maps of hypoxia- and inflammation-related genes using machine learning. **(D)** PCA shows that the visualization of data based on PC1 and PC2 can be clearly distinguish between patients with ICH at different stages. **(E)** Visualizes the ability to distinguish patients with ICH at different stages of development by tSNE method.

To evaluate the genes associated with hypoxia or inflammation that are closely related to ICH development, SVM–RFE and LASSO analyses were applied to screen DEGs. SVM–RFE showed minimal error in outcome prediction when all 31 hypoxia- or inflammation-related DEmRNAs were selected (Figure 3A). In a subsequent analysis, the Lasso analysis identified 13 hypoxia- or inflammation-related genes that were strongly associated with ICH development (Figure 3B). Figure 3C shows the co-expression network of these 13 genes, and *TIMP1* and *PLAUR* were found to be co-expressed with several genes. Moreover, PCA and tSNE visualizations demonstrate that these 13 genes can be used to distinguish patients with ICH at different stages (Figures 3D,E).

### Venn Analysis Identified Four DEGs Associated With Inflammation or Hypoxia

**Figure 4:**
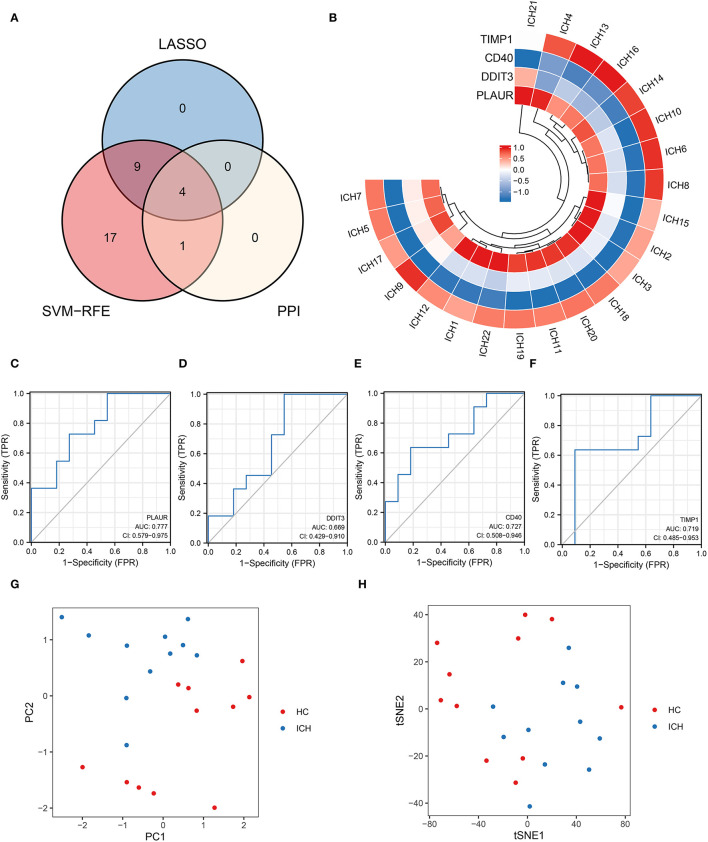
Four key genes obtained from the cross-tabulation analysis. **(A)** The Venn diagram shows that four core genes are shared in the PPI network, SVM–RFE, and LASSO. **(B)** Relative expression profiles of these four core genes in all the patients. **(C)** PALUR, **(D)** DDIT3, **(E)** CD40, and **(F)** the ability of TIMP1 to differentiate between patients with ICH at different development stages. **(G)** PCA and **(H)** tSNE visualization showing the ability to discriminate between patients with ICH at different development stages.

An intersection analysis of hypoxia- or inflammation-related DEGs obtained from machine learning and PPI networks was performed, and four core inflammation- or hypoxia-related DEGs (*TIMP1, PLAUR, DDIT3*, and *CD40*) were identified (Figure 4A). The relative expression profiles of these four core genes in patients with ICH are shown in Figure 4B. The ROC curves for *PLAUR* (AUC = 0.777), *DDIT3* (AUC = 0.669), *CD40* (AUC = 0.727), and *TIMP1* (AUC = 0.719) for the different stages of ICH development are shown in Figures 4C–F, respectively, indicating their moderate predictive power. PCA and tSNE visualization showed that patients with different stages of ICH can be distinguished based on these four genes (*TIMP1, PLAUR, DDIT3*, and *CD40*) (Figures 4G,H). Therefore, four biomarkers (TIMP1, PLAUR, DDIT3, and CD40) were screened for their association with inflammation or hypoxia by machine learning.

### Transcription Factors and Corresponding miRNAs That Regulate *TIMP1*

**Figure 5:**
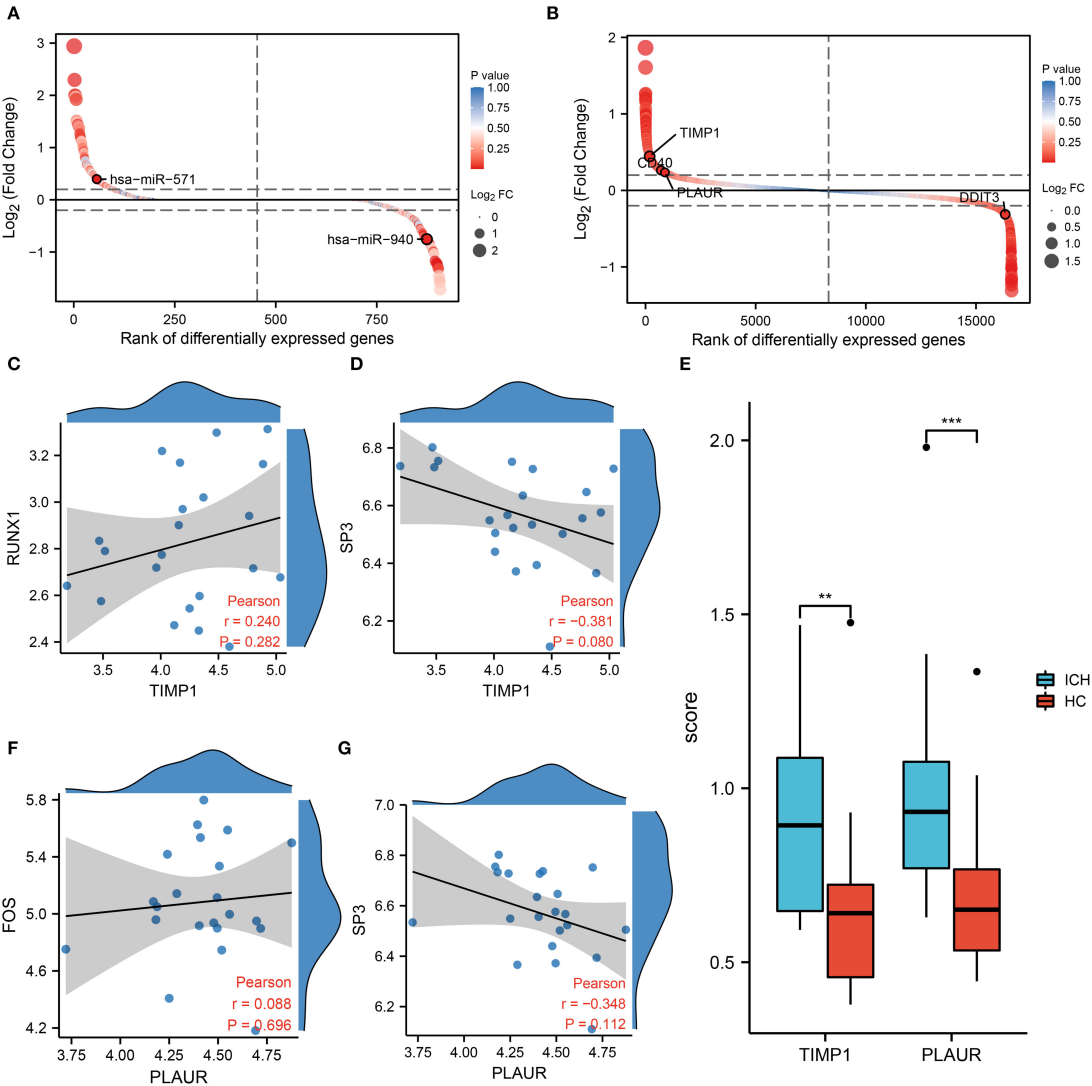
Results of co-expression analysis of four core genes. DEmiRNAs **(A)** DEmRNAs **(B)** are arranged by log_2_ (Fold Change). **(C)** Correlation analysis between *RUNX1* and the inflammation-associated gene *TIMP1*. **(D)** Correlation analysis between *SP3* and the inflammation-associated gene *TIMP1*. **(E)** PCR showed differential expression of *TIMP1* and *PLAUR* between the HC and ICH groups. **(F)** Correlation analysis between FOS and PLAUR. **(G)** Correlation analysis between SP3 and PLAUR.

In the regulatory network, we discover that RUNX1 and SP3 may act as transcriptional regulators of *TIMP1* (Figure 2C). RUNX1 is regulated by hsa-miR-940, and SP3 is regulated by hsa-miR-571 (which has the most junctions). The magnitudes of the fold change values for hsa-miR-940 and hsa-miR-571 are shown in Figure 5A. The relative ranking of the fold change values for *TIMP1, PLAUR, DDIT3*, and *CD40* are shown in Figure 5B. Further correlation analysis showed a positive correlation between *RUNX1* and *TIMP1* (*r* = 0.24, Figure 5C) and a negative correlation between *SP3* and *TIMP1* (*r* = −0.381, Figure 5D). Although there was no statistically significant difference, more cellular research are needed. Therefore, we further predicted two miRNA–TF–mRNA axes around the inflammation-related gene TIMP1, namely, hsa-miR-940/RUNX1/*TIMP1* and hsa-miR-571/SP3/TIMP1 (Figure 2C). Validation in an independent clinical cohort showed statistically significant differences in *TIMP1* expression between ICH and HC (*p* < 0.01, Figure 5E).

### Transcription Factors and Corresponding miRNAs That Regulate PLAUR

In the molecular interaction network (Figure 2C), the transcription factors of the hypoxia-related gene *PLAUR* were SP3 and FOS. SP3 also exhibited a regulatory effect on the inflammation-related gene *TIMP3*. Based on a condition of >0.3 correlation coefficient, we found no co-expression between *FOS* and *PLAUR* (*r* = 0.08, *p* = 0.282, Figure 5F), while there was some negative correlation between *SP3* and *PLAUR* (*r* = −0.348, Figure 5G). Therefore, we identified SP3 as a key hypoxia- and inflammation-related transcription factor. In addition, hsa-miR-571/SP3/*PLAUR* was constructed around *SP3* and *PLAUR* as an axis miRNA-TF-mRNA (Figure 2C). There was a significant difference in *PLAUR* expression between ICH and HC based on the data from an independent clinical cohort (*p* < 0.01, Figure 5E).

### SP3 Is Involved in Leukocyte- and Platelet-Related Physiological Processes

**Figure 6:**
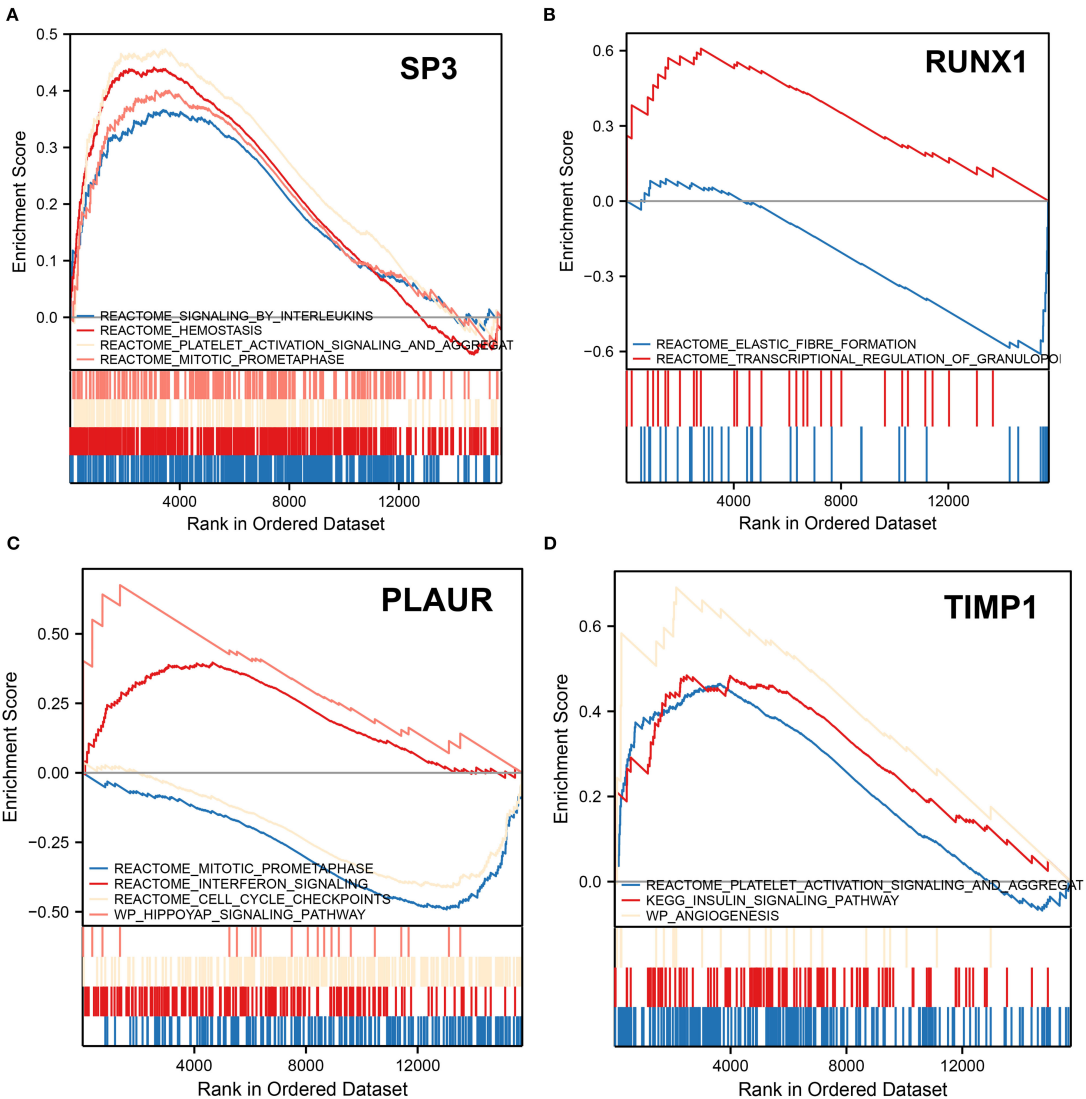
Gene set enrichment analysis. **(A)** GSEA shows altered pathways in samples with downregulated SP3. **(B)** GSEA shows altered pathways in samples with upregulated RUNX1. **(C)** GSEA shows altered pathways in samples upregulated by PLAUR. **(D)** GSEA shows altered pathways in samples with TIMP1 upregulation. The upward peaks of the curve represent the upward adjustment pathway, while the downward peaks represent the downward adjustment pathway.

We performed GSEA analysis of the transcription factors SP3 and RUNX1 to explore the pathways in which they might be involved. GSEA showed that the signaling by interleukins, hemostasis, platelet activation signaling and aggregation, and mitotic prometaphase pathways were upregulated in patients with ICH with SP3 downregulation (Figure 6A). In patients with ICH with elevated RUNX1 expression, elastic fiber formation was downregulated, while the transcriptional regulation of the granulopoiesis pathway was upregulated (Figure 6B). The aforementioned results indicate that SP3 may be involved in interleukin signaling and platelet activation for hemostasis, while RUNX1 may be involved in granulopoiesis after ICH onset.

### *TIMP1* Is Associated With Platelets and Angiogenesis

In patients with ICH with *PLAUR* upregulation, mitotic prometaphase and cell cycle checkpoints were downregulated, while interferon signaling and hippoyap signaling pathway were upregulated (Figure 6C). In patients with ICH with elevated expression of *TIMP1*, the platelet activation signaling and aggregation, insulin signaling pathway, and angiogenesis pathways were upregulated (Figure 6D). *PLAUR* and *TIMP1* were both upregulated 3 days after ICH onset. Therefore, it is hypothesized that *PLAUR*, a hypoxia and inflammation-related gene, may be involved in upregulating the interferon signaling pathway and hippo pathway, while *TIMP1* may be involved in platelet activation and aggregation, activating insulin signaling pathways, and promoting angiogenesis.

## Discussion

Four hypoxia- or inflammation-related biomarkers (*TIMP1, PLAUR, DDIT3*, and *CD40*) were identified in this study. Among them, SP3 might act as a transcriptional regulator for *TIMP1* and *PLAUR*. A bioinformatics approach was used to predict the possible roles of the three hypoxia- and inflammation-related miRNA–TF–mRNA axes (hsa-miR-940/RUNX1/*TIMP1*, hsa-miR-571/SP3/*TIMP1*, and hsa-miR-571/SP3/*PLAUR*) in ICH development. Independent clinical cohort studies have validated upregulation of *TIMP1* and *PLAUR* expression after the onset of ICH. In addition, GSEA was used to analyze the functions of *SP3, RUNX1, TIMP1*, and *PLAUR*.

*TIMP1* and *PLAUR* have been identified in the previous studies as involved in the progression of ICH. Matrix metalloproteinases (MMPs) are the most important degrading enzymes in the pathogenesis of ICH (49), during extracellular matrix reconstruction and blood–brain barrier disruption (50). TIMP1 is a major endogenous inhibitor of MMP-9 and was found to be significantly more expressed in the serum of patients with ICH than in normal controls in a study of the Chinese Han patients with ICH (51). TIMP-1 expression is also associated with early mortality in ICH as its potential biomarker for predicting mortality (52). The urokinase-type fibrinogen activator encoded by the *PLAUR* gene plays an important role in the development of cortical neural circuits and in brain tissue remodeling after brain injury (53, 54). Thus, *PLAUR* can be related to the prognosis of patients with ICH. In this study, *TIMP1* and *PLAUR* were upregulated 3 days after the onset of ICH compared with that before the onset. Previous studies have found that TIMP1 is associated with primary sarcopenia, colon cancer progression and metastasis, and some infectious diseases (55–57). We found that TIMP1 is involved in platelet activation and aggregation, insulin signaling pathway activation, and angiogenesis. The upregulation of *TIMP1* can therefore affect recovery, regression, and progression of the patients with ICH by affecting both platelet function and angiogenesis. And *PLAUR* is associated as an inflammation-related gene with diseases or processes, such as asthma, myocardial infarction, and reduced lung function (58–60). TIMP1 and PLAUR are involved in inflammation and hypoxia-related progression in ICH. MMP-9 is expressed in inflamed tissues and is involved in the inflammatory process (61). TIMP-1 is a natural inhibitor of MMP-9 (62). Thus, TIMP-1 might play a role in the inflammatory response by inhibiting MMP-9. After inflammation occurs, PLAUR binds to PLAU and activates plasminogen to plasmin, promoting inflammatory cell migration and activation and matrix metallopeptidase (MMP) activation, thereby participating in the inflammatory response (60, 63–65). In addition, PLAUR can be regulated by the hypoxia-inducible factor HIF-1 to play a role in the hypoxia-related mechanisms of the disease (66). In this study, TIMP1 and PLAUR upregulation in ICH was validated in an independent cohort, which confirms the involvement of TIMP1 and PLAUR in ICH development.

In this study, we found that both *TIMP1* and *PLAUR* are regulated by SP3, a transcription factor associated with both hypoxia and inflammation. Hypoxia has long been found to downregulate SP3 (67). The SP transcription factor family can be involved in the regulation of hypoxic gene expression in the hippocampus through a mechanism mediated by oxidative stress during hypoxia (68). In addition, SP3 is involved in the molecular regulatory mechanism of hypoxia-inducible factor 1α (69). Following inflammatory stimuli, SP3 and NF–κB interact to regulate inflammatory gene expression (70). SP3 is also involved in LPS-induced cellular inflammation (71). In patients with concomitant SP3 downregulation, signaling by interleukins, hemostasis, platelet activation signaling and aggregation, and mitotic prometaphase pathways were upregulated. These pathways suggest that downregulated SP3 is involved in interleukin signaling, platelet activation, and hemostasis. Therefore, SP3 downregulation might influence the progression and regression of ICH by affecting the degree of platelet activation as well as the level of inflammation, ultimately affecting the prognosis and quality of life of patients.

The miRNA–TF–mRNA network identified hsa-miR-571 as the pivotal miRNA regulating SP3. Previous studies have found that miR-571 functions in DNA replication and genomic stability (72). miR-571 is involved in the inflammatory process in cirrhosis (73), and can regulate the activation of human stem stellate cells (74) by mediating the Notch3 signaling pathway (75). However, there is a lack of studies on the relationship between miR-571 and SP3. In this study, we found the first evidence suggesting that hsa-miR-571 regulated the level of inflammation and platelet activation in ICH by regulating SP3 translation. And hsa-miR-571/SP3/TIMP1 and hsa-miR-571/SP3/PLAUR are two miRNA-TF-mRNAs involved in ICH development.

TIMP1 and PLAUR were differentially expressed in ICH and were upregulated 3 days after the onset of ICH. The upregulation of TIMP1 might have influenced the outcome of patients with ICH by affecting the platelet function and angiogenesis. PLAUR, in turn, was involved in the upregulation of the interferon signaling pathway and the hippo pathway. The hypoxia- and inflammation-related transcription factor SP3 was involved in the regulation of TIMP1 and PLAUR. SP3 might have influenced the progression of ICH by affecting the degree of platelet activation and the inflammation level. These findings provide potential targets for the diagnosis, treatment, and regression of ICH in order to monitor the severity of inflammation and hypoxia in patients with ICH. Although clinical samples were used for validating the study results, the number of clinical samples was small and the strength of validation needs improvement. Bioinformatic findings will need to be validated in the relevant cell lines as well as ICH animal models in the future. In addition to investigate the relationship between SP3 and its counterparts hsa-miR-571, *TIMP1*, and *PLAUR*, more research is needed to identify the role of SP3 in ICH.

## Conclusion

The hsa-miR-940/RUNX1/TIMP1, hsa-miR-571/SP3/TIMP1, and hsa-miR-571/SP3/PLAUR play important roles in ICH development. The hypoxia- and inflammation-related transcription factor SP3 might be involved in platelet activation in ICH through the regulation of TIMP1/PLAUR, as well as in inflammatory regulation.

## Data Availability Statement

The original contributions presented in the study are included in the article/Supplementary Material, further inquiries can be directed to the corresponding author/s.

## Ethics Statement

The studies involving human participants were reviewed and approved by General Hospital of Ningxia Medical University's Ethics Committee. The patients/participants provided their written informed consent to participate in this study.

## Author Contributions

DW, JF, PW, ZY, and TS performed the data curation and analysis. DW, JF, and TS analyzed and interpreted the results. TS drafted and reviewed the manuscript. All authors read and approved the final manuscript.

## Conflict of Interest

The authors declare that the research was conducted in the absence of any commercial or financial relationships that could be construed as a potential conflict of interest.

## Publisher's Note

All claims expressed in this article are solely those of the authors and do not necessarily represent those of their affiliated organizations, or those of the publisher, the editors and the reviewers. Any product that may be evaluated in this article, or claim that may be made by its manufacturer, is not guaranteed or endorsed by the publisher.
