# Supplementary material for: Hypoxia- and Inflammation-Related Transcription Factor SP3 May Be Involved in Platelet Activation and Inflammation in Intracranial Hemorrhage
Source: Front Neurol. 2022 Jun 2;13:886329. doi: 10.3389/fneur.2022.886329 (PMC9201407; doi:10.3389/fneur.2022.886329)
Supplement: Supplementary file 1 [file Data_Sheet_1.DOC]

| Supplementary table. Worksheet of public datasets | | |
| --- | --- | --- |
| Datasets | Summary | DATABASE LINK |
| GSE43618 | Transcriptomic data from patients with ICH | [https://www.ncbi.nlm.nih.gov/geo/query/acc.cgi?acc=GSE43618](https://www.ncbi.nlm.nih.gov/geo/query/acc.cgi?acc=GSE33000) |
| GSE125512 | Patients with ICH at onset and three days after onset | [https://www.ncbi.nlm.nih.gov/geo/query/acc.cgi?acc=GSE125512](https://www.ncbi.nlm.nih.gov/geo/query/acc.cgi?acc=GSE44770) |
